# A Pilot Study of the Effect of Deployment on the Gut Microbiome and Traveler’s Diarrhea Susceptibility

**DOI:** 10.3389/fcimb.2020.589297

**Published:** 2020-12-15

**Authors:** Blake W. Stamps, Wanda J. Lyon, Adam P. Irvin, Nancy Kelley-Loughnane, Michael S. Goodson

**Affiliations:** ^1^ 711th Human Performance Wing, Airman Systems Directorate, Air Force Research Laboratory, Wright-Patterson AFB, OH, United States; ^2^ Integrative Health and Performance Sciences Division, UES Inc., Dayton, OH, United States

**Keywords:** gut microbiome, 16S rRNA gene, diarrhea, dysbiosis, deployment

## Abstract

Traveler’s diarrhea (TD) is a recurrent and significant issue for many travelers including the military. While many known enteric pathogens exist that are causative agents of diarrhea, our gut microbiome may also play a role in TD susceptibility. To this end, we conducted a pilot study of the microbiome of warfighters prior to- and after deployment overseas to identify marker taxa relevant to TD. This initial study utilized full-length 16S rRNA gene sequencing to provide additional taxonomic resolution toward identifying predictive taxa.16S rRNA analyses of pre- and post-deployment fecal samples identified multiple marker taxa as significantly differentially abundant in subjects that reported diarrhea, including *Weissella*, *Butyrivibrio*, *Corynebacterium*, uncultivated Erysipelotrichaceae, *Jeotgallibaca*, unclassified Ktedonobacteriaceae, *Leptolinea*, and uncultivated Ruminiococcaceae. The ability to identify TD risk prior to travel will inform prevention and mitigation strategies to influence diarrhea susceptibility while traveling.

## Introduction

Traveler’s diarrhea (TD) is a recurrent and serious issue for many travelers abroad including the military. TD is the most prevalent illness during deployment, and military populations have experienced TD while on deployment since records began (Connor and Farthing, 1999). Deployed populations experience up to 36.3 cases of TD per 100 person-months, with the highest incidence rate during the first month of travel (Olson et al., 2019). Indeed, TD, combined with other disease, non-battle injuries, has a significant adverse impact on military operations, resulting in more hospitalizations and lost person-days than combat casualties (Sanders et al., 2005). TD is commonly ascribed to pathogenic bacteria such as *Salmonella*, *Campylobacter jejuni*, and *Escherichia coli* (including enterotoxigenic, enteroaggregative, and enteropathogenic strains); however, 40% of reported cases of TD have no known etiology (Olson et al., 2019). One possible explanation for the unknown or unclassified etiology of TD is a disruption of the collective gut microbiota, or gut microbiome. There is evidence that the gut microbiome plays a role in TD, whether that role is in the manifestation of diarrhea or in allowing for currently unknown pathogens to colonize and result in TD, and more broadly in that TD can change the gut community structure (David et al., 2014).

The human gut microbiome encompasses thousands of bacterial, eukaryotic, and archaeal species (Huttenhower et al., 2012). A number of human ailments includes an altered or aberrant microbiome. Daily sampling and community analysis revealed how profoundly our gut can be altered by infection as well as diet change during travel (David et al., 2014); however, this study relied on a small number of participants. Military populations are vulnerable to a multitude of stressors including sleep disruption, psychological stress, circadian disruption, significant alterations to diet, and environmental stressors, all of which likely contribute to alterations in the gut microbiota (Karl et al., 2018).

While identification of specific pathogens is certainly of interest, within military populations or any traveler, understanding if a specific microorganism or microbiome population can predict susceptibility or resilience to diarrhea is potentially more desirable to prevent the problem before it could become an impediment to performance. Previously, a study within the US Air Force (USAF) at an air base in Honduras identified microbial taxa that were potentially predictive of the development of TD within the study population (Walters et al., 2020). However, in this study, samples were collected once the population was deployed and all subjects were deployed to a singular location.

To expand upon previous deployment associated TD work, we initiated a pilot study of the gut microbiome of warfighters both prior to, and after deployment at a multitude of locations across the globe. While the number of microbiome samples is small, this pilot study offers a view into the effect of deployment and TD on the microbiome of warfighters through the use of surveys and a sheared 16S rRNA gene sequencing approach to generate new hypotheses and identify potentially predictive microbial biomarkers.

## Materials and Methods

### Sampling and Survey of Deployment Associated Diarrhea

Potential subjects were briefed about the study and were consented during their pre-deployment briefing. Subjects that consented to fecal sampling were asked to provide samples before and after deployment. Additionally, after deployment, at their post-deployment briefing, individuals were also asked to answer a short survey (**Supplemental Figure S1**) similar to that in our previously published work (Walters et al., 2020) related to their broad deployment location (also known as unified combatant commands), dietary choices, diarrhea before, during, and after deployment, and the number of episodes of diarrhea during each of these three phases. Deployment locations were defined as AFRICOM (Africa), CENTCOM (Middle East), EUCOM (Europe), INDOPACOM (Indo-Pacific region), NORTHCOM (North America), and SOUTHCOM (South America). For dining choices, subjects were asked if their primary source of food was the base dining facility (DF), local vendor food (LV), or meals ready to eat (MRE, **Supplemental Figure S1** and **Table S1**). Data were gathered, de-identified, and then recorded digitally with no record of subject name or other identifiable information.

The BBL CultureSwab EZ II Collection and Transport System (Becton, Dickenson and Company, Sparks, MD) was utilized to directly collect a small amount of fecal material after a bowel movement commensurate with the protocol employed by the American Gut Project (McDonald et al., 2018). Collection tubes containing samples were returned within 24 h of the bowel movement and were frozen at -80 °C. This study was approved by the Institutional Review Board at the 711th Human Performance Wing, Air Force Research Laboratory at Wright Patterson Air Force Base in Ohio, protocol number FWR20150052.

### DNA Extraction and Sequencing Library Preparation

DNA was extracted from swabs using the QIAamp DNA Microbiome kit (QIAGEN, Germantown, MD) according to manufacturer’s instructions. The 16S rRNA gene V2-V8 hypervariable region were amplified using PCR with primers S-D Bact 0008 c-S-20 (5’ AGRGTTYGATYMTGGCTCAG3’) and S-D Bact 1391- a-A-17(5’GACGGGCGGTGWGTRCA’3) (Klindworth et al., 2013). Briefly, a 50-μl reaction was set up using AccuStart II PCR Supermix (Quantabio, Beverly, MA) per the manufacturer’s instructions, including 10 ng of template DNA. Polymerase chain reaction (e.g., amplicon) cycling parameters were as follows: 3 min at 95°C, then 25 cycles of 30 s at 95°C, 30 s at 55°C, and 30 s at 72°C, with a final extension at 72°C for 5 min. Amplicons were visualized by Invitrogen E-gel agarose gel electrophoresis, and then purified using Agencourt AMPure XP beads at a final concentration of 1.8 X (Beckman Coulter, Brea, CA). Final amplicon concentration was quantified using Qubit dsDNA high sensitivity assay (Life Technologies). Amplicon sequencing libraries were then prepared using an Illumina Nextera XT Library preparation kit (Illumina, San Diego, CA), followed by sequencing PE150 sequencing on a Illumina MiSeq.

### Data Analysis

Sequence reads were assigned initial taxonomy using Kraken 2 (Wood and Salzberg, 2014) with the SILVA database (Quast et al., 2013) serving as a reference. After initial assignment taxonomy was further refined using Bracken (Lu et al., 2017) for each sample. Individual Bracken reports were then imported into Pavian (Breitwieser and Salzberg, 2020) before a taxonomy table containing all samples was exported as a tab delimited file. This was then imported into Phyloseq within R (McMurdie and Holmes, 2013; R Core Team, 2013) to aid visualization and statistical testing. Alpha diversity of samples was assessed using the packages “DivNet” and “Breakaway” (Willis et al., 2017; Willis and Martin, 2020). Ordinations were generated within AmpVis2 (Andersen et al., 2018). A Dirichlet Multinomial Mixture Model was also produced to estimate the number of metacommunities within the dataset (Holmes et al., 2012) using the microbiome R package. Differential abundance and variability was assessed using COunt RegressioN for Correlated Observations with the Beta-binomial, or Corncob (Martin et al., 2019).

## Results

**Figure 1:**
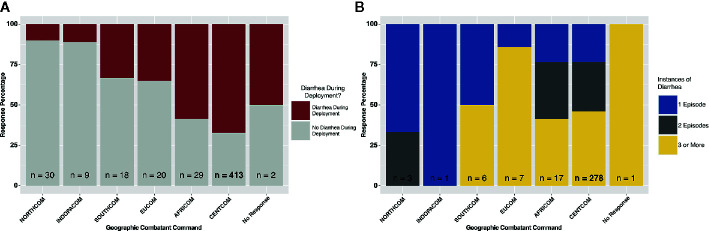
Deployment diarrhea survey summary statistics. **(A)** “yes/no” response grouped by geographic combatant command, and **(B)** for those that responded “yes”, the number of instances of diarrhea on deployment, again grouped by combatant command.

A total of 521 subjects responded to survey questions, and 23 provided fecal samples either prior to or after deployment, or both. Thirteen subjects provided fecal samples both before and after deployment (hereafter referred to as “linked” samples). Subject numbers were heavily skewed toward those that deployed to the CENTCOM region (n = 413), while all other deployment regions reported fewer than 30 respondents each. Pre-deployment diarrhea rates ranged from 0% (INDOPACOM) to 20% (EUCOM). The largest cohort, CENTCOM, reported pre-deployment diarrhea rates of 9% (**Table 1**). Subjects reporting diarrhea on deployment ranged from 10% in North America (NORTHCOM) to 67% in CENTCOM (**Figure 1A**). Of those reporting diarrhea, multiple instances were common, again mostly within CENTCOM (**Figure 1B**). Notably, 46% of respondents with diarrhea reported three or more instances while on deployment, with 29% and 24% of respondents reporting two or one instance of diarrhea on deployment, respectively. Timing of TD also varied, with 42% reporting their first case of diarrhea on deployment within the first 2 weeks, 32% at 5 weeks or more while on deployment, and 26% reporting their first instance of diarrhea on deployment between 2 and 5 weeks. Of those that experienced diarrhea on deployment, 96% reported bottled water as their only drinking water source, while 62% reported solely eating at a base dining facility. Additional survey results are available in supplemental data (**Supplemental Table S1**). A total of 52% of subjects that donated feces reported diarrhea on deployment, in line with survey statistics.

**Table 1:** Summary statistics of survey respondent numbers for each combatant command, diarrhea either before, during, or after deployment, and the percentage of respondents whom ate primarily at the deployed base dining facility.

Deployment Command	Respondent Number	Pre-Deployment Diarrhea	Deployment Diarrhea	Post- Deployment Diarrhea	DF as Primary Eating Location
AFRICOM	29	7%	59%	14%	52%
CENTCOM	413	9%	67%	16%	60%
EUCOM	20	20%	35%	15%	5%^a^
INDOPACOM	9	0%	11%	0%	0%^b^
NORTHCOM	30	7%	10%	0%	6%^c^
SOUTHCOM	18	17%	33%	11%	33%
No Response	2	0%	50%	0%	100%^d^

^a^40% of respondents consumed local vendor (LV) foods.

^b^Majority of respondents (55%) consumed mix of dining facility (DF) and LV foods.

^c^43% of respondents consumed LV foods.

^d^Only two subjects failed to respond to deployment location.

**Figure 2:**
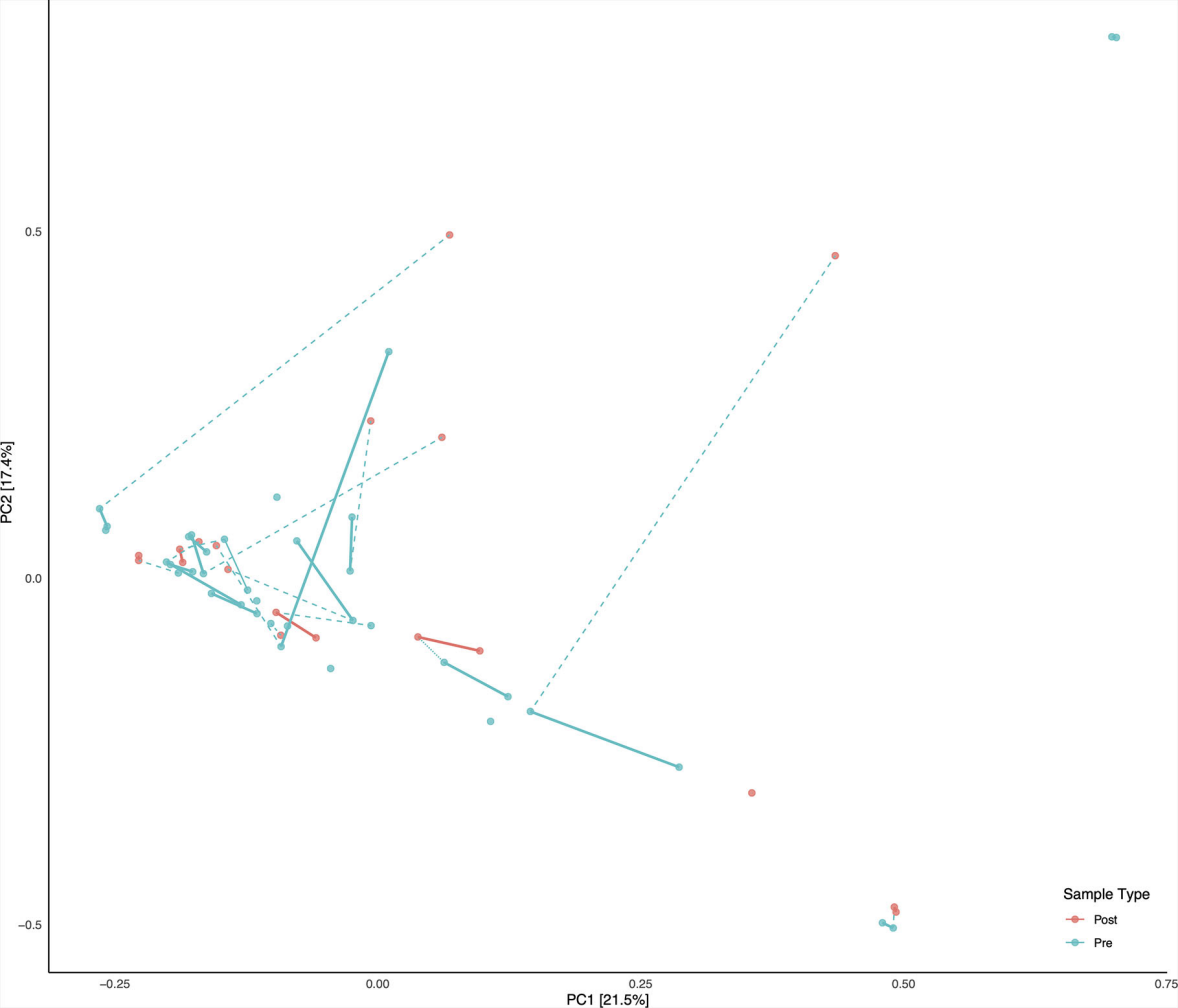
Principal Component Analysis (PCA) of all samples. Subjects with linked pre/post samples are shown with dashed lines connecting the pre and post sample types. Solid bars connect the same sample types (Pre or Post) within each subject on the PCA.

**Figure 3:**
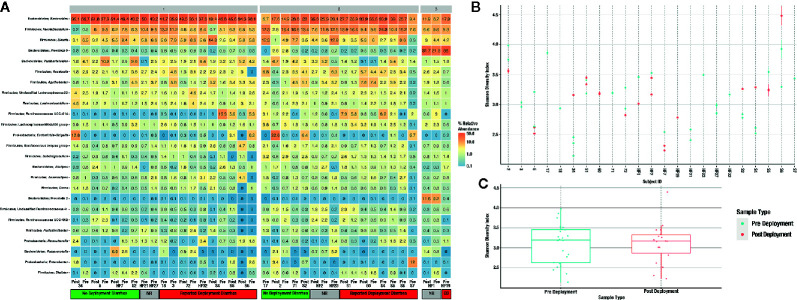
Heatmap of the 25 most abundant taxa, clustered by genus **(A)**. Samples are faceted by DMM metacommunity, and by whether the respondents were diarrheal prior to, and while on deployment. Subjects that gave no response to the questionnaire are noted as “unknown” within the heatmap. Observed alpha diversity by individual subject **(B)**, and average alpha diversity by deployment status **(C)**, both estimated by the Shannon diversity index.

A total of 56 fecal samples were collected from the cohort of 23 subjects with 37 collected prior to deployment and 19 after return from deployment. All 56 samples were successfully sequenced resulting in 5.5 million sequence reads. Median library size was 103,994 reads after quality control and taxonomy assignment. Individual library size and metadata can be found in **Supplemental Table S2**. Overall there was a non-significant difference between pre- and post-deployment samples (ADONIS p > 0.05) although two subjects with linked pre- and post-deployment samples, Subjects 2 and 34, had large shifts in community membership (**Figure 2**). Otherwise, subjects with linked pre- and post- deployment samples (Subjects 5, 51, 60, 72, HF1, 2, and 7, and S2, 4, 5, and 6) had little to no change in their overall community composition as estimated by a Bray Curtis distance matrix and visualized by PCoA (**Figure 2**). After assignment of samples into metacommunities by DMM, general trends of community membership emerged (**Figure 3A**). These included a high Bacteroidetes relative abundance group (Metacommunity 1), an intermediate Bacteroides group (Metacommunity 2), and a low Bacteroides/high Prevotella group (Metacommunity 3). Subjects 2 and 34 had an opposite shift in the relative abundance of the *Escherichia/Shigella* present (**Figure 2**) before or after deployment, with a notable decline from 12.8 to 0% for subject 34, and an increase from 0.1% to 22.6% relative abundance for subject 2. Neither subject experienced diarrhea on deployment. Many subjects remained within the same metacommunity before and after deployment, although several (S2, S4, S5, S6) shifted between a high and intermediate *Bacteroides* relative abundance (metacommunities 1 and 2) (**Figure 3A**). Subject alpha diversity was variable, with some subjects increasing in estimated Shannon diversity after deployment and many having a decrease in estimated diversity (**Figure 3B**), but in the aggregate Shannon diversity was lower, but not significantly different after deployment (p > 0.05, **Figure 3C**). This is supported by a low number of significantly differentially abundant taxa (17, **Supplemental Figure S3B**) before or after deployment. These included *Lactobacillus*, *Pantoea*, *Cornybacterium*, *Megamonas*, *Brenneria*, and *Anaeroplasma.* However, when samples were tested for differential abundance of taxa by those that reported diarrhea on deployment, 32 taxa were differentially abundant toward those subjects reporting diarrhea (**Figure S3A**). The majority of these taxa were very low relative abundance (**Supplemental Figure S2A, B**); however, one taxon, Ruminococcaceae UCG-014, was both differentially abundant in those with diarrhea during deployment and above 3% relative abundance on average in all sampled communities prior to deployment (**Figure 3A**). Other strongly differential taxa included *Weissella*, *Butryvibrio*, *Brenneria*, *Buchnera*, and *Sutterella* (**Figure 3A**). (While significantly differentially abundant taxa were observable between sample grouping, both by diarrhea status (yes vs. no) on deployment and by deployment status (pre-vs. post deployment), abundances of most differential taxa were largely invariant within a sample group. For example, there was low variability of differentially abundant taxa within the “diarrhea status yes” group (**Supplemental Figure S4**).

## Discussion

TD impacts over half of individuals during deployment, representing a detriment to personal comfort, health, and the ability to carry out mission objectives. A recent comprehensive review of TD literature by Olson et al. (2019) suggests that acute TD is still prevalent among not just the military but the broader American population as well. While known pathogenic microbiota are responsible for the majority of reported TD cases, greater than 40% still have no known cause (Olson et al., 2019). Whether this is due to emergent infectious enteric pathogens, a dysbiosis of the microbiome directly causing TD, or a dysbiosis allowing an unknown pathogen to induce TD remains to be identified. Still, any ability to predict if certain individuals may be more susceptible than others to TD would greatly improve the performance of military travelers and also improve the quality of life of all individuals traveling abroad by ensuring individuals take more care in food choices, administration of prophylactic TD medicines, and more broadly by being more aware of their individual risks for diarrhea while abroad. This work identified several potential marker taxa worthy of additional study in the future, with more comprehensive deep metagenomic sequencing studies. Further, we have shown the need for sampling not just after diarrhea or other negative effects have occurred, but in collection of samples prior to deployment.

Our survey data confirms previous findings of high percentages of TD while deployed (Olson et al., 2019). Almost half of all respondents that experienced diarrhea while on deployment reported 3 or more cases of diarrhea. Despite this, 60% of respondents in CENTCOM (where the majority of subjects deployed) reported the on-base dining facility as their primary source of food, suggesting sources other than food may be the cause of reported diarrhea. Further, 90% of all subjects also reported that bottled water was their only source of drinking water. While variations in the microbiome could be the cause of the reported TD, other potentially less complicated sources of the discrepancy between reported food and water intake and the high level of TD could also be the case. Our survey was only given after deployment, and likely suffers from recall bias as subjects may of incorrectly recalled what food items they consumed, where their water came from, or the number of diarrheal episodes they had (Althubaiti, 2016). Further selection bias may have occurred because of personnel who were medically evacuated and not returned to duty because of a severe illness or injury would likely not have been available for this survey, potentially resulting in an underestimate of the impact of illness and injury. Special operations units or task forces, which may have been exposed to especially austere or dangerous conditions, may have had separate transportation capabilities and could be under-represented. Still, the survey data represents a useful source of TD data from a deployed population. And while known pathogens may be the cause of TD, the microbiome may also play a role.

With the exception of two subjects, there were few apparent differences in subject microbiomes before and after deployment when visualized by principal component ordination (**Figure 2**). This suggests that despite high levels of TD while on deployment, most subjects had few changes in their gut microbiome that persisted after deployment was complete. This is consistent with data from a recent longitudinal deployment microbiome study (Walters et al., 2020). Instead and with few exceptions, prior to, and/or after, deployment the subjects were separated into three distinct metacommunities identified by utilizing Dirchelet Multinomial Mixture modeling (**Figure 3A**). Largely these communities were influenced by two major gut bacterial genera, the *Bacteroides* and the *Prevotella*. Notably, *Prevotella* were largely absent within the two other major communities (**Figure 3**). Previous large-scale analyses have noted that *Prevotella* exist as one end-member (with *Bacteroides* as the other) of a gradient of human gut microbiomes, rather than discrete enterotypes (Jeffery et al., 2012). Our work appears to support this assertion. Overall the literature is mixed in the potentially dys- or eubiotic properties of *Prevotella* (Precup and Vodnar, 2019). However, increased relative abundances of *Prevotella* are associated with increased risk of diarrheal irritable bowel syndrome, or IBS-D, while individuals with high relative abundances of *Bacteroides* were not (Su et al., 2018). Within this study only two subjects were within the *Prevotella* dominated community, and only one completed a survey, but did report diarrhea while on deployment.

Focusing instead on subjects whom reported diarrhea status while on deployment and also donated linked samples before and after deployment, we can identify several microbial taxa of interest for further study. Differential abundance analyses were used to determine which, if any, microbiota may be linked causally to predict if a subject were to develop or be protected from diarrhea during deployment. Limiting the focus to those taxa identified as being more than two fold differentially abundant, seven taxa were associated with a lack of diarrhea, including the Enterobacteriaceae *Buchnera*, *Brenneria*, and *Plesiomonas*; as well as *Sutterella*, Prevotellaceae Ga6A1, Erysiplotrichaceae UCG-004, and *Anaeroplasma*. Both *Buchnera* and *Brenneria* are more associated with plants, either as obligate endosymbionts or as pathogens with little to no known association with the human gut (Douglas, 1998; Octavia and Lan, 2014). *Plesiomonas* was previously associated as a potential, emerging enteric pathogen (Theodoropoulos et al., 2001) and so its’ association within our dataset with subjects that did not develop diarrhea requires further study. Members of the Prevotellaceae, specifically the *Prevotella* are associated with vegetarian or vegetable rich diet (Precup and Vodnar, 2019). The specific Prevotellaceae we identified, Ga6A1, was previously associated with cellulose rich diets in waterfoul (Guo et al., 2019). The Erysiplotrichaceae have conflicting associations, and while increased relative abundances of this family have been associated with gut inflammation, the opposite has also been found (Kaakoush, 2015).

Taxa that were increased in subjects reporting TD included *Weissella*, *Butyrivibrio*, *Corynebacterium*, uncultivated Erysipelotrichaceae, *Jeotgallibaca*, unclassified Ktedonobacteriaceae and *Leptolinea*, *Weissella*, and *Butyrivibrio* were previously reported as either beneficial (via production of butyrate) or as potential probiotics so their increased abundance is curious, although literature related to *Weissella* is unsettled on its potential pathogenicity, and of interest in further study (Abriouel et al., 2015; Vital et al., 2017). The Erysipelotrichaceae are highly immunogenic and may contribute to gut inflammation and/or gastrointestinal issues (Kaakoush, 2015). Both the Ktedonobacteriaceae and *Leptolinea* are within the Chloroflexi, a highly diverse lineage adapted to a multitude of diverse aquatic and terrestrial environments including the mammalian gut, although at a low relative abundance (Ley et al., 2008). While not more than two-fold differentially abundant, members of the Bacteroidetes included an increase in abundance of the genus *Coprobacter* in subjects that reported diarrhea during deployment. *Coprobacter* was previously associated with a Chinese population that consumed a largely high fat diet (Qian et al., 2018) but, to date, we could find no prior association of *Coprobacter* with any specific disease status.

While most of these differentially abundant taxa were not high in relative abundance (**Figure 3**, **Supplemental Table S3**), one relatively high abundance (up to 15.3% relative abundance in one subject) unclassified genus within the *Ruminococcaceae* family was identified (**Figure 3A**). *Ruminococcaceae* UCG-014 relative abundance was differentially abundant in subjects that reported TD during deployment (**Supplemental S3A**). *Ruminococcaceae* UCG-014 has been previously described in mouse model studies of neuropathic pain and in cattle microbiome surveys, but to this point never associated as a potential predictive marker of diarrhea (Yang et al., 2019; Zhang et al., 2019). Within metacommunity 1, UCG-014 was more abundant prior to deployment; however, in metacommunity 2 this pattern was flipped and a greater relative abundance of the genera was found in post deployment samples. Still, it could be that the lineage occupies a fundamental niche in the guts of those predisposed to TD. Other unclassified Ruminococcaceae genera have been recently associated with TD, specifically Ruminococcaceae UCG-013 which was differentially abundant in a deployed military population in Honduras (Walters et al., 2020). Further work will confirm the role unclassified Ruminoccocaceae play in the development of diarrhea, but their presence could predispose those in metacommunity 2 to TD in future deployments. There is certainly anecdotal evidence that gastrointestinal issues follow multiple TD episodes during deployment, but this correlation requires additional study. Future, larger investigations involving deployed populations will attempt to further elucidate the connection between *Ruminococcaceae* UCG-014 and the risk of diarrhea, if any.

It should be noted that our study has limitations, which were expected as a pilot study. The number of independent subjects enrolled in the microbiome was limited due to difficulties with recruitment of active military personnel, especially during a period of upheaval associated with deploying. Further, age and other demographic data were also not able to be collected. A much larger cohort of individuals will be required to confirm our correlative observations and to build more accurate predictive models that allow for the assignment of relative risk of TD susceptibility. Subjects volunteered to participate, and as such, this created a bias toward individuals who are willing to participate in survey and fecal sampling studies. The consenting period (and paperwork) occurred within the larger context of pre deployment briefings which may have discouraged additional subjects from enrolling. Further, upon return fewer subjects gave samples, possibly due to a lack of interest in continuing the study after deployment. Additionally, the results may be context dependent; the microbial signature that indicates protection or susceptibility to TD may not apply to non-US populations and, within US populations, the same gut microbial community may not be indicative of susceptibility or protection from TD if challenged by an environment with a different microbial milieu than those that the majority of our respondents were exposed to in the Middle East (for example, in SE Asia where TD-causing *Campylobacter* spp. predominate).

The identification of microbial risk factors associated with TD remains of crucial interest to military populations worldwide. Our work represents a first step toward larger, ongoing investigations of deployed military populations Caution is warranted in distilling these results to provide actionable information. The number of subjects that completed both pre and post sampling was small (n = 13) limiting the power of the study to produce definitive conclusions. Future studies will include larger cohorts and follow their performance and diet prior to, during, and after deployment to more conclusively identify microbiota correlated to diarrheal status, with particular interest toward the Ruminococcaceae. This preliminary study suggests that several taxa may be predictive of diarrhea status prior to travel. The ability to identify TD risk prior to travel has benefits beyond the military and will inform prevention and mitigation strategies for the comfort of any traveler in the future, including modulation of the gut microbiome using prebiotic, probiotic, or synbiotic methods.

## Author’s Note

This manuscript has been released as a pre-print at bioRxiv (Stamps et al., 2020).

## Data Availability Statement

The original contributions presented in the study are publicly available. This data can be found here: https://www.ncbi.nlm.nih.gov/bioproject/?term=prjna659594.

## Ethics Statement

The studies involving human participants were reviewed and approved by Institutional Review Board 711th Human Performance Wing, Air Force Research Laboratory. The patients/participants provided their written informed consent to participate in this study.

## Author Contributions

BS conducted the analyses and prepared the manuscript. MG conceptualized the experiment, conducted sampling, and edited the manuscript. WL generated the sequencing libraries and edited the manuscript. AI conducted sampling and edited the manuscript. NK-L conceptualized the experiment and edited the manuscript. All authors contributed to the article and approved the submitted version.

## Funding

This work was made possible by funding from the 711th Human Performance Wing Research, Studies, Analyses, and Assessment Committee and the Defense Health Program Joint Program Committee-5 Working Group.

## Conflict of Interest

BS is an employee of UES, Inc.

The remaining authors declare that the research was conducted in the absence of any commercial or financial relationships that could be construed as a potential conflict of interest.

## Supplementary Material

Supplementary Figure 1 Example of survey given to study participants.

Supplementary Figure 2 Heatmap of significantly differentially abundant taxa **(A)** in subjects with traveler’s diarrhea during deployment and significantly differentially abundant taxa in subjects before and after deployment **(B)**.

Supplementary Figure 3 Differentially abundant taxa in subjects with linked (pre/post deployment) samples, either comparing subjects with or without diarrhea on deployment **(A)** or those taxa differentially abundant before and after deployment **(B)**. Only taxa found to be significantly differentially abundant after false discovery rate error correction are shown in the figure.

Supplementary Figure 4 Differential variability of taxa in subjects with linked (pre/post deployment) samples, either comparing subjects with or without diarrhea on deployment **(A)** or those taxa differentially abundant before and after deployment **(B)**. Taxa shown are only those found to be differentially abundant in **Supplemental Figure S3**.

Supplementary Table 1 Detailed survey responses.

Supplementary Table 2 Mapping file including read numbers per library.

Supplementary Table 3 Per sample taxonomy table after merger in Pavian.
