# Supplementary material for: A Pilot Study of the Effect of Deployment on the Gut Microbiome and Traveler’s Diarrhea Susceptibility
Source: Front Cell Infect Microbiol. 2020 Dec 15;10:589297. doi: 10.3389/fcimb.2020.589297 (PMC7770225; doi:10.3389/fcimb.2020.589297)
Supplement: Supplementary Figure 1 — Example of survey given to study participants. [file DataSheet_1.pdf]

Deployment-associated diarrhea survey

This voluntary survey is designed to gather information about the incidence and burden of diarrhea before, during, and after deployment to overseas locations. For this study, ***diarrhea is defined as three or more loose stools in a 24-hour period***. No personally identifiable information is requested or required. This survey has been reviewed by the 711th Human Performance Wing Institutional Review Board.

1) What country were you deployed to? ______________________________

Before deployment

2a) Had you experienced diarrhea in the 3 months before your deployment? Yes ☐ No ☐

2b) If yes, how many different episodes did you experience? 1X ☐ 2X ☐ >3X ☐

During deployment

3a) Did you experience diarrhea during your deployment? Yes ☐ No ☐

3b) When during your deployment did you experience diarrhea? Weeks: 1-2 ☐ 3-4 ☐ 5+ ☐

3c) How many different episodes did you experience during deployment? 1X ☐ 2X ☐ >3X ☐

3d) How did you treat your symptoms? No treatment ☐ Over-the-counter ☐ Antibiotics ☐

3e) How many duty days were lost as a result of the diarrhea? 0 ☐ 1 ☐ 2 ☐ 3 ☐ 4+ ☐

3f) Did you only drink bottled water? Yes ☐ No ☐

3g) Where did the food you ate come from? Dining facility ☐ MRE ☐ Local vendors ☐

After deployment

4a) Have you experienced diarrhea since returning from deployment? Yes ☐ No ☐

4b) How many episodes have you experienced since returning? 1X ☐ 2X ☐ >3X ☐
