# Supplementary figures and images for: A Pilot Study of the Effect of Deployment on the Gut Microbiome and Traveler’s Diarrhea Susceptibility
Source: Front Cell Infect Microbiol. 2020 Dec 15;10:589297. doi: 10.3389/fcimb.2020.589297 (PMC7770225; doi:10.3389/fcimb.2020.589297)

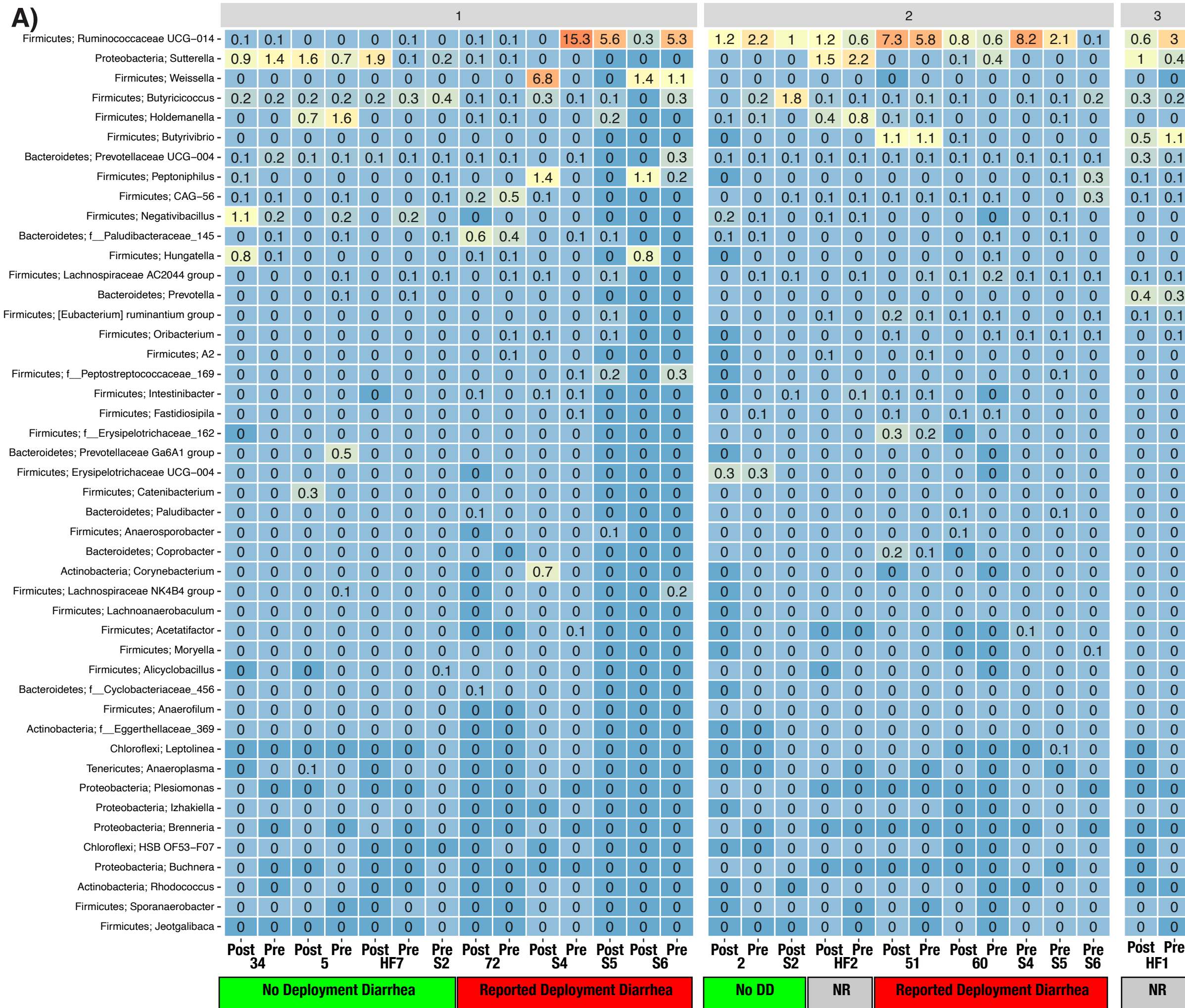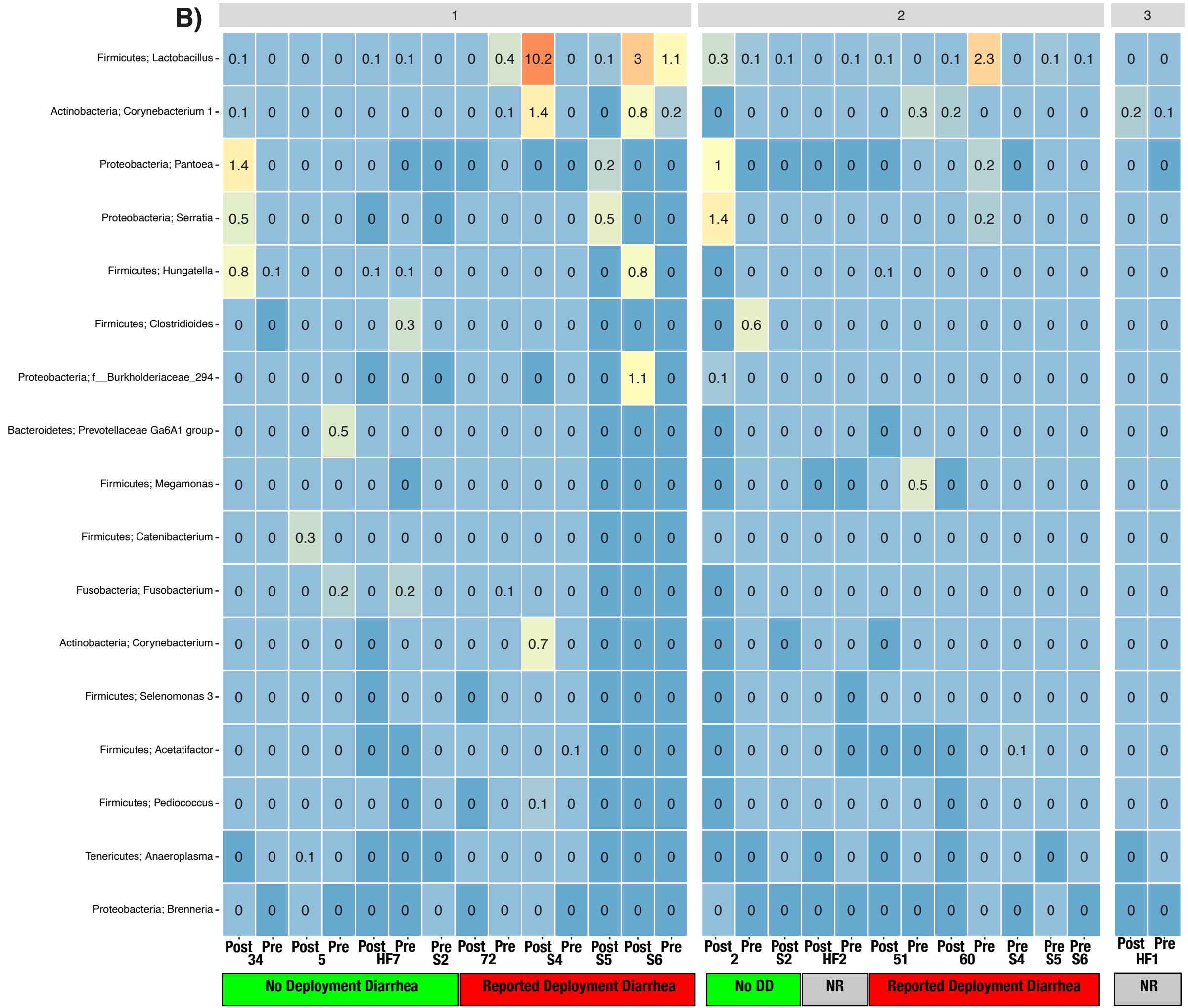

Supplement: Supplementary Figure 2 — Heatmap of significantly differentially abundant taxa (A) in subjects with traveler’s diarrhea during deployment and significantly differentially abundant taxa in subjects before and after deployment (B). [file DataSheet_2.pdf]

A)

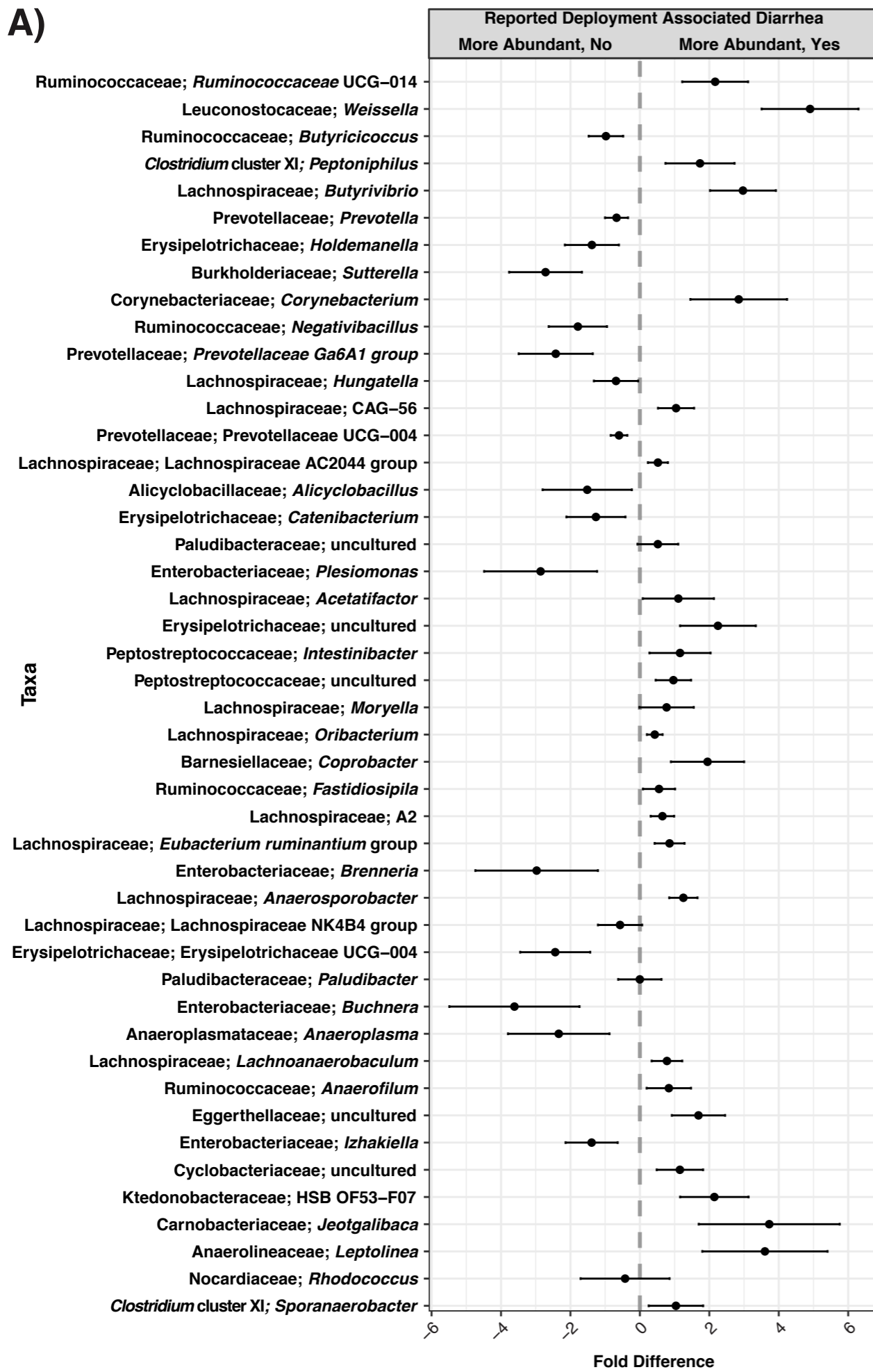

B)

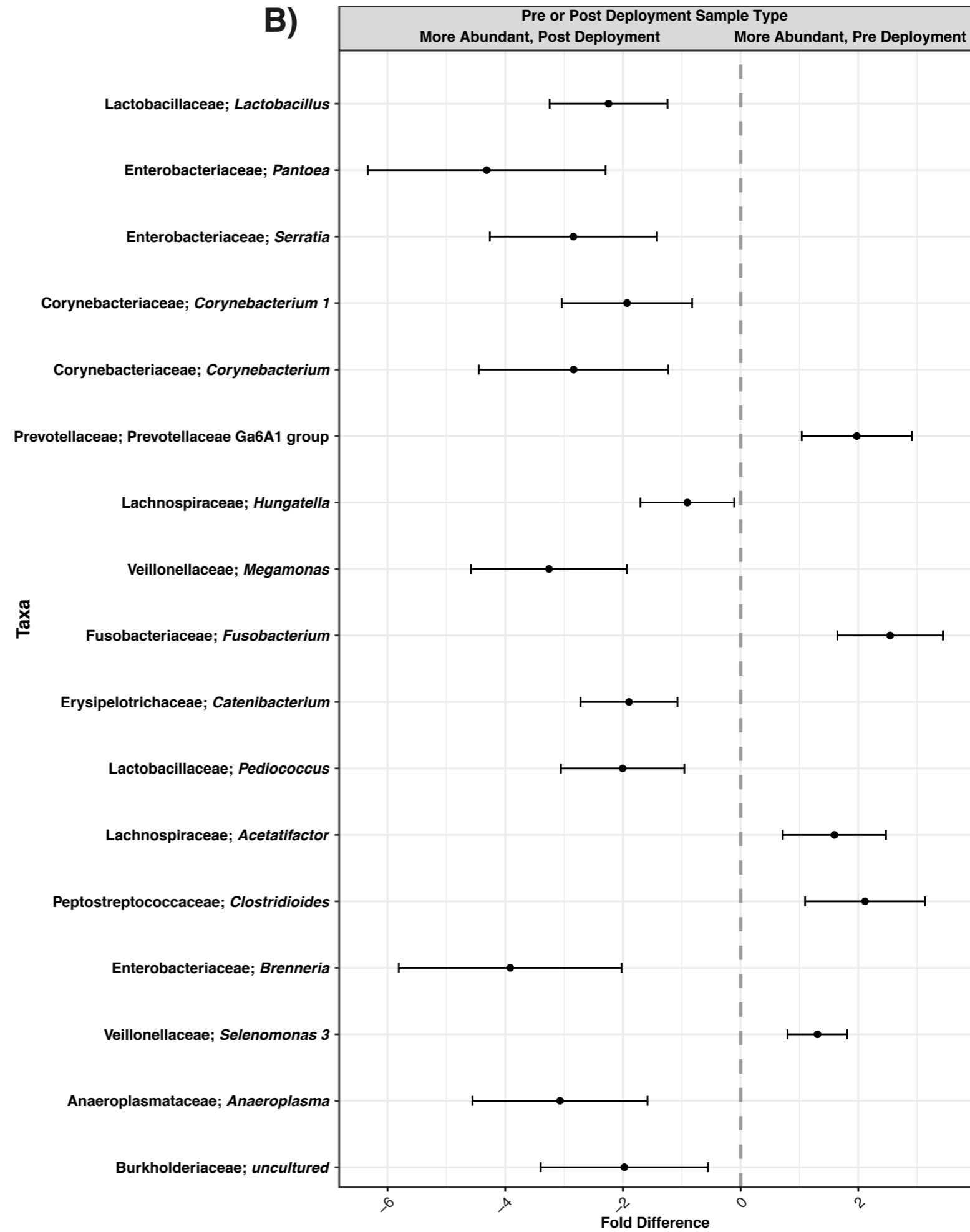

Supplement: Supplementary Figure 3 — Differentially abundant taxa in subjects with linked (pre/post deployment) samples, either comparing subjects with or without diarrhea on deployment (A) or those taxa differentially abundant before and after deployment (B). Only taxa found to be significantly differentially abundant after false discovery rate error correction are shown in the figure. [file DataSheet_3.pdf]

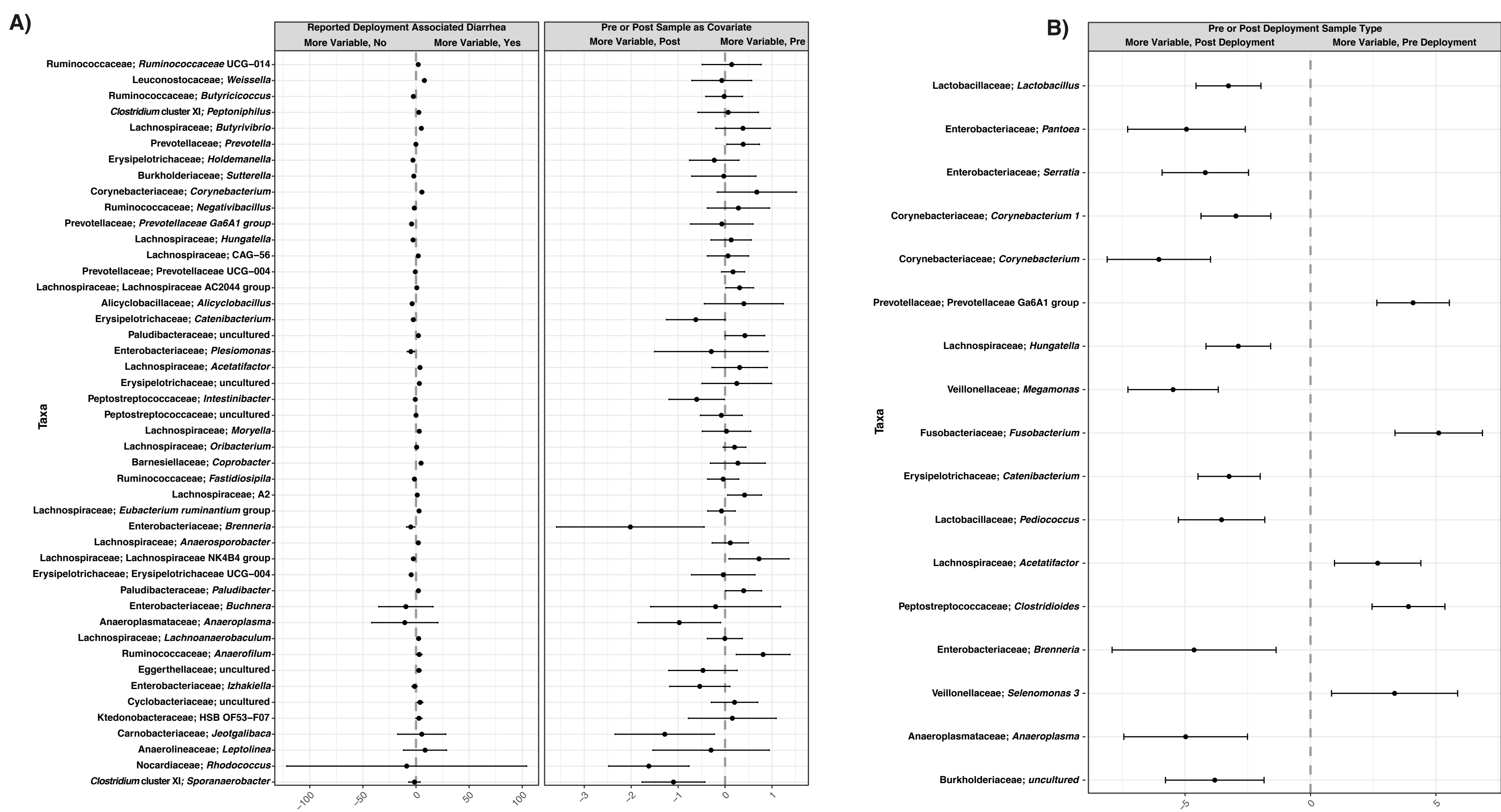

Supplement: Supplementary Figure 4 — Differential variability of taxa in subjects with linked (pre/post deployment) samples, either comparing subjects with or without diarrhea on deployment (A) or those taxa differentially abundant before and after deployment (B). Taxa shown are only those found to be differentially abundant in Supplemental Figure S3 . [file DataSheet_4.pdf]
